# Functional node layout in Metroidvania game worlds: A complex network analysis

**DOI:** 10.1371/journal.pone.0354705

**Published:** 2026-07-31

**Authors:** GL(Guanglong) Bao, Beihe Weng, Long Ding, Qianjin Du, Yemin Zang, Jiarui Wang, Fei Yan

**Affiliations:** Art and Design College, Yangzhou University, Yangzhou, Jiangsu, China; Universiti Malaya, MALAYSIA

## Abstract

This study investigates how the spatial structure of Metroidvania game worlds relates to the layout of key functional nodes. Using Hollow Knight, Prince of Persia: The Lost Crown, and Blasphemous 2 as case studies, the maps were transformed into graph structures based on spatial data compiled from gameplay observation, in-game maps, community maps, and verification through gameplay videos. On the basis of this graph representation, the study adapts the Nearest Neighbor Index by replacing Euclidean distance with network shortest-path distance in order to evaluate the distribution patterns of save points, teleport points, and boss points. It further employs multi-source k-hop reachability analysis to measure service coverage and redundancy. Finally, closeness centrality and betweenness centrality are calculated on the full network, and Spearman rank correlation is used to test the association between functional areas and centrality. The results reveal clear differences in the structural roles of functional nodes across the three games. In Hollow Knight and The Lost Crown, save points are relatively dispersed, weakly coupled with centrality, and function as a distributed safety net. In Blasphemous 2, save points exhibit higher small-radius coverage and redundancy and are more strongly aligned with central positions, indicating a hub-like stronghold structure. Teleport systems likewise reflect different design strategies, ranging from exploration-oriented sparse layouts to later-stage mobility optimization. Boss points in all three games generally remain structurally isolated, showing low overlap and weak centrality association. These findings suggest that differences in the placement of save points, teleport points, and boss points reflect distinct structural design strategies, and that network analysis can provide a quantitative basis for evaluating and optimizing such layouts in level design.

## 1. Introduction

Video game space, as a distinctive form of virtual environment, has become an important object of study at the intersection of game studies, architectural studies, and urban studies [1,2]. Players’ understanding of game worlds is formed through processes of exploration and reorientation [3–5], during which paths, boundaries, connections, and hierarchical relations are gradually recognized, remembered, and organized. Accordingly, game space can be understood as a structural system that organizes action, perception, and experience, with its core characteristics lying in its rule-driven discrete organization and navigable structural properties [4,5]. These features also establish a methodological connection between video game space and configurational analysis in architecture and urban studies, allowing game space to be further abstracted as a topological system composed of nodes and connections.

In architectural and urban studies, spatial network analysis has been widely used to identify structural centers, assess accessibility, and examine the relationship between spatial organization and functional distribution. Existing studies have shown that structural centers, accessibility differentials, and spatial hierarchies in street networks significantly influence the locational patterns and clustering characteristics of commercial facilities, public services, collection-and-delivery points, and other activity nodes [6–8]. At the same time, studies incorporating POI data further suggest that urban functions must be understood through street sequences, co-occurrence relations, and overall network structures, and that the formation and internal organization of functional areas should be examined through the spatial associations among different facilities [8–10]. This line of inquiry is equally relevant to Metroidvania games. As a genre characterized by exploration, gating, backtracking, and non-linear progression, the spatial experience of Metroidvania games is likewise shaped by the placement of key functional nodes within the overall structure [11].

From a methodological perspective, network-based and configurational studies of video game space remain relatively limited. Existing studies have already begun to treat game levels as spatial systems amenable to formal analysis. Taking Castle Wolfenstein 3D as an example, Martin distinguishes between the “map image” and “traversable space” as two analytical layers of the level, and combines visibility analysis and axial analysis to examine how spatial form shapes the player’s visual-motor experience through visibility distribution, path organization, visual depth, and integration, thereby discussing the mechanisms through which level form itself produces meaning [12]. Li’s study goes a step further by applying graph theory to non-linear level design, using minimal logic graphs, connectivity, and stability analysis to examine how the topological organization of non-linear levels affects navigation and spatial memory [13].

Despite this progress, two issues still warrant further investigation. First, although existing studies have begun to examine the network organization, topological relations, and path structures of video game spaces, they have focused primarily on explaining overall spatial form and navigation experience, while the distribution patterns, structural positions, and operating mechanisms of functional nodes within the broader configuration remain underexplored. Second, although research on real-world space has already developed a set of approaches for analyzing the relationship between spatial structure and functional facilities, these approaches still require adjustment when applied to video game studies in order to account for the characteristics of different game types.

On this basis, this study develops a network-analytic framework for Metroidvania game spaces. Using Hollow Knight, Prince of Persia: The Lost Crown, and Blasphemous 2 as case studies, it abstracts traversable relations on their maps into graph structures and examines the relationship between overall topology and three types of functional nodes: save points, teleport points, and boss points.

The contributions of this study are twofold. First, the study treats the layout of functional nodes as an independent analytical object in the study of game space and proposes a quantitative method tailored to functional node layouts in Metroidvania game spaces. This framework integrates network-adapted nearest-neighbor analysis, k-hop coverage and redundancy analysis, and centrality correlation analysis, thereby systematically characterizing the layout features and structural relationships of save points, teleport points, and boss points. Second, through a comparative analysis of three representative cases, the study reveals the differentiated structural roles played by different types of functional nodes in game worlds. The study further demonstrates that network analysis can not only explain the overall organizational characteristics of game space, but also help identify problems in functional node layouts, such as uneven coverage, excessively high or low service overlap, overly peripheral placement, or a failure to effectively fulfill their intended structural roles, thereby providing quantitative support for optimizing functional layouts in level design.

## 2. Materials

### 2.1. Case studies and data sources

This study selects Hollow Knight (2017), Prince of Persia: The Lost Crown (2024), and Blasphemous 2 (2023) as its core case studies for the following reasons. First, all three belong to the Metroidvania genre and share a broadly comparable spatial logic characterized by exploration, ability gating, backtracking, and non-linear progression, which provides a common basis for comparison. Second, all three games feature relatively complete systems of functional nodes, including save points, teleport points, and boss points, thereby offering stable and comparable cases for the analysis of functional node layouts. For this study, we utilized official game maps, player-made guide maps, in-game map functions, and spatial information obtained through practical gameplay by researchers. In some instances, potentially incomplete or imprecise game spatial connection data were used—for example, path networks spontaneously drawn or annotated by player communities—which may suffer from insufficient completeness or missing details. To mitigate this limitation, we incorporated spatial exploration records shared on game forums and player communities, as well as live gameplay videos from streaming platforms as important supplements.

### 2.2. Spatial modeling

Spatial network modeling of video games is the process of abstracting the game environment into a network structure. This process is inherently a theoretical act, as Butts pointed out: “representing an empirical phenomenon as a network is a theoretical act… the appropriate choice of representation is crucial for obtaining correct results [14].” In this study, this abstract modeling process requires careful selection of representation methods. This includes generating a simplified representation of the spatial network by selecting key research elements and identifying the relationships between them. Crucially, it determines what will be represented as nodes (vertices) and links (edges) in the graph, as well as which additional parameters of the spatial network the graph should capture. This step is termed network modeling [15]. As discussed earlier, game space networks can also be subjects of complex network research, and their modeling approach fundamentally influences the research outcomes. This similarity allows us to draw on methods from related disciplines while making necessary adjustments tailored to the specificities of game spaces. Based on this, the definitions of nodes and edges in this study are as follows:

#### 2.2.1. Nodes: Spatial units.

In this study, a spatial unit in a platform game is defined as a region that can be identified by the player at the map level and that has clear boundaries. The boundaries of spatial units are determined by the entry or exit points of scene transitions. Specifically, this study uses the standard scene-transition mechanism in the games, namely the screen fade-in/fade-out transition, as the basis for segmentation: whenever the player passes through a transition point and enters a new map region, it is identified as a new spatial unit. All three case-study games—Hollow Knight, Prince of Persia: The Lost Crown, and Blasphemous 2—use this scene-transition method. If an apparently separate scene is not treated as an independent region in the game’s map logic, for example, if it is only a small room within an existing area and has no separate map marker, it is incorporated into its parent spatial unit and is not identified as a new node.

#### 2.2.2. Edges: Connectivity relationships.

In this study, the connection between nodes is defined by their passability. Passability refers to whether the connection between adjacent spatial units allows the player’s avatar to move from one unit to another. These spaces need not be connected in the typical physical sense. A connection is considered impassable only if direct passage between the connected units is perpetually disallowed. Passability is specifically defined for the player’s avatar, as the player is the agent performing navigation; a connection that other characters can use but the player character can never pass through is considered impassable [16].

### 2.3. Functional units: Functional points

A functional unit is a spatial unit endowed with specific interactive functions, defined as follows:

#### 2.3.1. Teleport point.

In this study, a teleport point is defined as a spatial unit equipped with a fast-travel function. As a rapid transit mechanism in video games, the teleporter constitutes a gamified practice analogous to the “jump cut” in film—a mechanism that replaces continuous spatial navigation with a discrete mode of travel [17]. This allows players to transcend the constraints of physical space and achieve instantaneous movement between different locations.

#### 2.3.2. Save point.

A “save point” in this paper refers to a spatial unit specifically designed to provide a save function. F.L. Geerts, in defining and classifying save mechanisms and analyzing their impact on game experience and narrative, notes that save points are a variant of manual saving—locations within the game where players can save. Typically, if this method is used, players cannot save freely at will via a menu but only at these designated locations in the game. This somewhat restricts when the user can save and completely limits where saving can occur [18]. In the three games analyzed in this study, only the save points in Blasphemous 2 not only provide a save function but also allow movement between different save points. Even so, they are treated here as a mechanism distinct from dedicated teleport points, because the two differ substantially in their network connectivity, accessibility conditions, and actual logic of use. This functional distinction may be compared to independently operated airline route networks: although both provide spatial connectivity, they differ significantly in network structure, node accessibility, and conditions of use. Such a distinction between mechanisms has an important influence on the sequences of play and the spatial experiences formed by the player.

#### 2.3.3. Boss point.

A “Boss Point” in this study is defined as a spatial unit specifically designed for triggering and engaging in combat with a Boss enemy. Bosses are generally understood as unique characters or creatures that are more complex and challenging than common enemies, with their encounters carefully placed at critical junctures in the game’s progression [19].

### 2.4. Rationale and considerations for choosing an undirected graph model

Although game environments often contain mechanisms for one-way connections (such as one-way portals or areas accessible only after meeting certain level requirements), this study employs an undirected graph model for analysis, primarily based on the following rationale: Firstly, the study focuses on the “centrality potential” inherent in the spatial layout itself. It aims to isolate the constraints of specific game mechanics to analyze the connectivity and accessibility of the underlying structure in its ideal state, thereby revealing the space’s intrinsic potential for facilitating player behavior and interaction. Secondly, using an undirected graph helps simplify the model and highlight the main topological features, allowing for clearer identification of structural centers. It also establishes an extensible baseline framework for potentially introducing directional weights or dynamic mechanisms in future work.

## 3. Methods

### 3.1. Nearest neighbor method

The nearest neighbor method was employed to analyze the spatial distribution patterns of functional nodes. Owing to its effectiveness in quantifying the distribution characteristics of point features, this method has been widely applied in related studies [20,21]. By comparing the observed mean nearest neighbor distance among point features with the expected distance under a random distribution assumption, the method can be used to determine whether a set of points exhibits clustered, random, or dispersed spatial patterns.

The core metric of this approach is the Nearest Neighbor Index (NNI), which is defined as the ratio of the observed mean nearest neighbor distance to the expected mean nearest neighbor distance under spatial randomness:


$$\begin{array}{c} \text{NNI}=\frac{{\overset{―}{D}}_{\text{obs}}}{{\overset{―}{D}}_{\text{exp}}} \end{array}$$ (1)

where the observed mean nearest neighbor distance is denoted by ${\bar{D}}_{\text{obs}}$, and the expected mean nearest neighbor distance under the random distribution assumption is denoted by ${\bar{D}}_{\text{exp}}$.

An NNI value significantly less than 1 indicates a clustered distribution, a value close to 1 suggests a random distribution, and a value significantly greater than 1 indicates a dispersed or uniform distribution pattern.

In this study, the traditional nearest neighbor index method was adapted to account for the networked nature of video game spaces. Specifically, network shortest-path distance was used in place of Euclidean distance. The distance between two nodes was defined as the number of edges along the shortest path connecting them in the graph, thereby more accurately reflecting actual accessibility constrained by traversable connections in the game space.

The analytical procedure consisted of two main steps:

(1) **Calculation of the observed mean nearest neighbor distance (**${\bar{D}}_{𝐨𝐛𝐬}$)

For a given category of target functional nodes (e.g., all save points), the shortest-path distance from each node to its nearest neighboring node of the same category was calculated. The observed mean nearest neighbor distance was then obtained by averaging these nearest neighbor distances across all target nodes:


$$\begin{array}{c} {\overset{―}{D}}_{\text{obs}}=\frac{\text{1}}{n}\sum_{i=1}^{n}{min}_{j\neq i}d\;\left(v_{i},v_{j}\right) \end{array}$$ (2)

where *n* denotes the number of target functional nodes, $v_{i}$ and $v_{j}$ represent nodes in the network, and $d(v_{i},v_{j})$ denotes the shortest-path distance between nodes $v_{i}$ and $v_{j}$.

(2) **Establishment of the random baseline (**${\bar{D}}_{𝐞𝐱𝐩}$)

To construct a robust random reference model, a Monte Carlo simulation with 1000 random samples was conducted. To eliminate the additional connectivity effects introduced by fast-travel mechanisms and to ensure that the random baseline more accurately reflects the underlying spatial configuration, the simulations were performed on a base network from which all dedicated teleport connections were removed.

In each simulation, a set of nodes equal in number to the target functional nodes was randomly selected from the base network, and the mean nearest neighbor distance was calculated. The average value across all simulations was taken as the expected mean nearest neighbor distance:


$$\begin{array}{c} {\overset{―}{D}}_{\text{exp}}=\frac{\text{1}}{M}\sum_{k=1}^{M}{\overset{―}{D}}_{\text{rand}}^{\left(k\right)},\text{\textbackslash{}hspace\{1em}\;\}M=1000 \end{array}$$ (3)

### 3.2. Service coverage and service redundancy analysis

To further examine how different types of functional nodes support player movement and progression within the network space, this study introduces a multi-source k-hop reachability analysis on the base network, that is, the network with dedicated fast-travel links removed. In level-design terms, this analysis is used to assess how effectively save points, teleport points, or boss points serve surrounding areas, how far their influence extends through traversable paths, and to what extent their service areas overlap or reinforce one another. Specifically, for a given category of target functional nodes, we treat them as a set of “source nodes.” Under a specified hop radius (k), we first identify which nodes in the base network can be reached by at least one source within that radius, and then quantify how many sources can reach each node within the same radius. Accordingly, for any node, we define its coverage count as the number of sources that can reach it within the given radius. The coverage count is defined as:


$$\begin{array}{c} c_{k}\left(v\right)=\sum_{s\in S}1(d\left(s,v\right)\leq k) \end{array}$$ (4)

where the shortest-path length is measured by the number of edges. When the coverage count is no less than one, the node is considered covered by the target set within that radius. Based on the coverage count, two core indicators are adopted in this study. The first is the coverage rate, which measures the proportion of nodes that can be reached within the given radius, defined as:


$$\begin{array}{c} Cov\left(k\right)=\frac{\left|\left\{v\in V:c_{k}\left(v\right)\geq 1\right\}\right|}{N} \end{array}$$ (5)

The second is service redundancy, which characterizes the degree of overlap in coverage. It is computed by averaging over all nodes, with uncovered nodes contributing zero, so that service overlap can be reflected on the same scale:


$$\begin{array}{c} Red\left(k\right)=\frac{\sum_{v\in V}c_{k}\left(v\right)}{N} \end{array}$$ (6)

A higher coverage rate indicates a larger service range of the given functional nodes at the k-hop scale, whereas a higher service redundancy generally means that more nodes can be reached by multiple sources within the same radius, i.e., stronger service overlap.

To determine whether the observed coverage rate and redundancy deviate significantly from a random layout, we further construct a Monte Carlo random baseline and conduct statistical tests. Specifically, for each analysis of a given functional-node category, we keep the number of sources equal to the number of target nodes. In each trial, the same number of nodes is randomly sampled without replacement from the full node set as a “random source set,” and the coverage rate and redundancy are recomputed under the same hop radii. This random sampling is repeated 1000 times to obtain the indicator distributions under randomness. Based on this, the z-score is used to quantify how the observed value deviates from the random expectation: z > 0 indicates that coverage/redundancy is higher than under a random layout, whereas z < 0 indicates that it is lower than under a random layout.

### 3.3. Statistical correlation analysis of functional areas and network centrality

To examine the statistical relationship between the topological environment of specific functional nodes (e.g., save points) and their centrality characteristics within the overall network, a three-step analytical procedure was adopted.

(1) **Definition of functional areas**

Based on the assumption that the influence of a functional node may extend to its immediately adjacent spaces, a functional influence area was defined for each category of functional nodes. This area includes all nodes possessing the target function (e.g., nodes with save functionality), as well as their first-order neighbors, that is, all nodes directly connected to them.

(2) **Centrality calculation on the complete network**

To evaluate the structural position of functional areas within the fully connected game space, centrality measures were computed on the complete network, which includes all traversable connections, including activated fast-travel links. Two core centrality metrics were calculated for each node: closeness centrality and betweenness centrality.

Closeness centrality is used to measure the global accessibility of a node within the network and is defined as the inverse of the sum of shortest-path distances from that node to all other reachable nodes:


$$\begin{array}{c} C_{c}\left(v\right)=\frac{\text{1}}{\sum_{u\neq v}d\left(v,u\right)} \end{array}$$ (7)

where d(v,u) denotes the shortest-path distance between node *v* and node *u*. Higher values indicate greater overall accessibility within the network.

Betweenness centrality is used to measure the extent to which a node acts as an intermediary or structural bottleneck in the network. It is defined as the sum of the proportions of all shortest paths that pass through the node:


$$\begin{array}{c} C_{B}\left(v\right)=\sum_{s\neq v\neq t}\frac{\sigma_{st}\left(v\right)}{\sigma_{st}} \end{array}$$ (8)

where $\sigma_{st}$ denotes the total number of shortest paths between nodes *s* and *t*, and $\sigma_{st}(v)$ denotes the number of those pa*t*hs that passthrough node *v*. Higher betweenness centrality values indicate greater structural importance in network flows.

(3) **Spearman rank correlation analysis**

To test the statistical association between functional areas and network centrality measures, a non-parametric Spearman rank correlation analysis was conducted. Specifically, a binary variable was constructed for each node to indicate whether it belongs to a given functional area (yes = 1, no = 0). Spearman correlation coefficients were then calculated between this binary variable and both closeness centrality and betweenness centrality values.

## 4. Results

**Fig 1:**
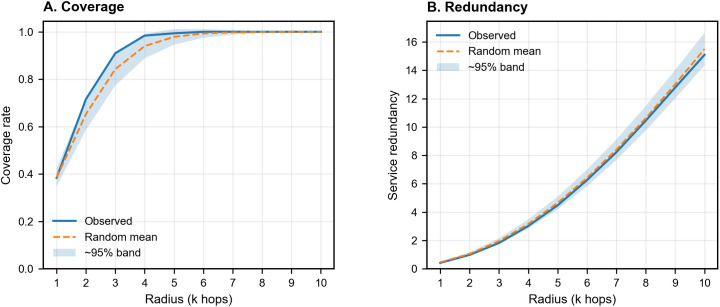
k-hop coverage and redundancy of save points in Hollow Knight.

**Fig 2:**
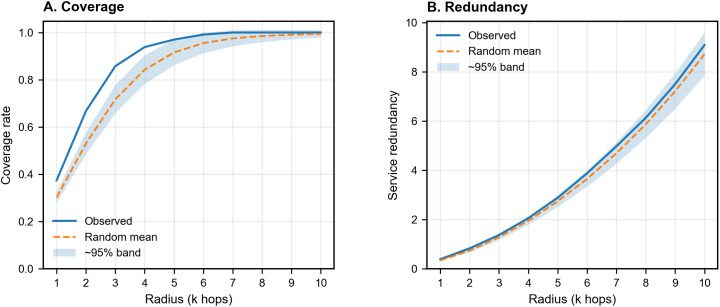
k-hop coverage and redundancy of save points in Blasphemous 2.

**Fig 3:**
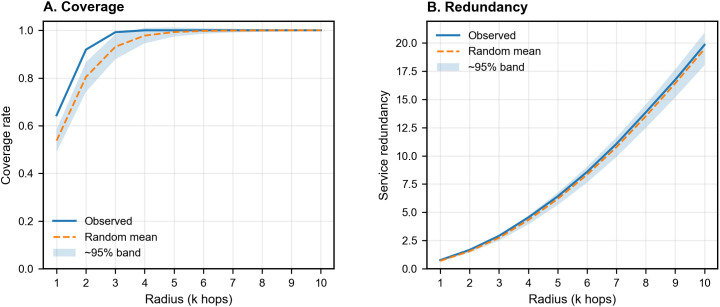
k-hop coverage and redundancy of save points in Prince of Persia: The Lost Crown.

**Fig 4:**
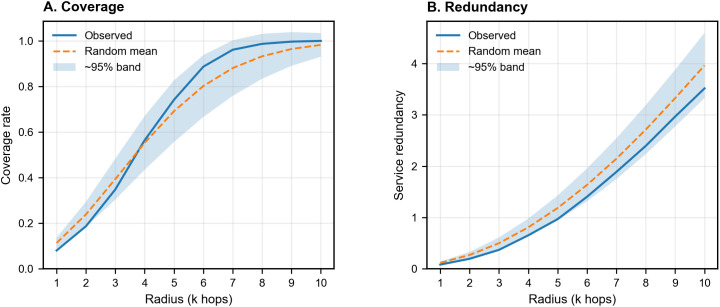
k-hop coverage and redundancy of teleport points in Hollow Knight.

**Fig 5:**
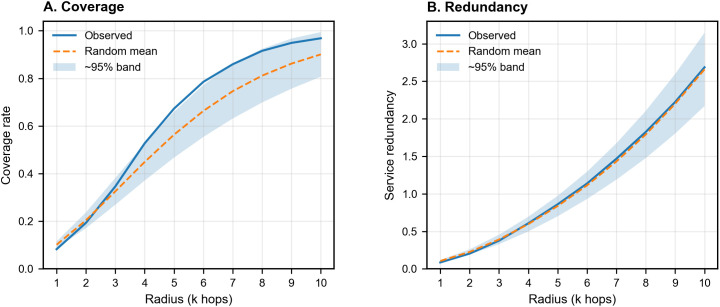
k-hop coverage and redundancy of teleport points in Blasphemous 2.

**Fig 6:**
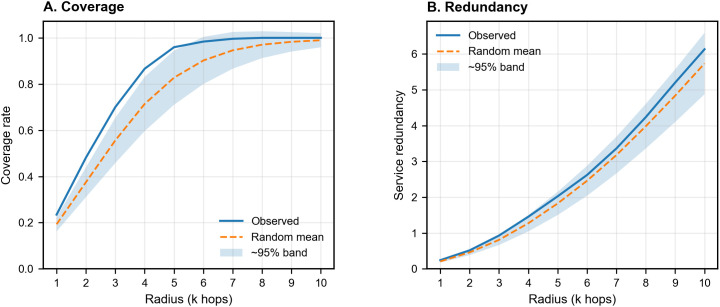
k-hop coverage and redundancy of teleport points in Prince of Persia: The Lost Crown.

**Fig 7:**
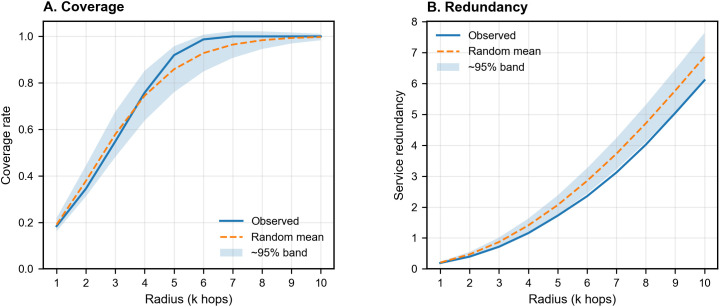
k-hop coverage and redundancy of Boss points in Hollow Knight.

**Fig 8:**
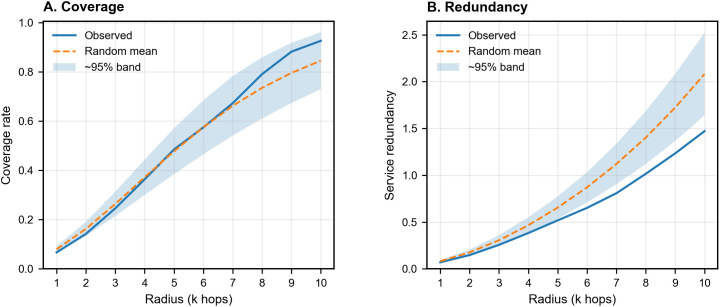
k-hop coverage and redundancy of Boss points in Blasphemous 2.

**Fig 9:**
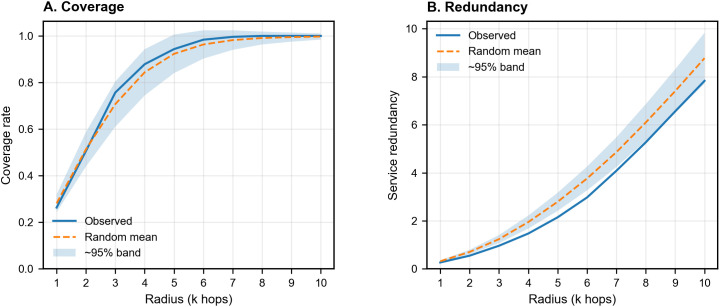
k-hop coverage and redundancy of Boss points in Prince of Persia: The Lost Crown.

Based on the methods described above, this section reports the quantitative results for the three types of functional nodes in the three case studies. The results are presented separately for save points, teleport points, and Boss points, and are discussed in terms of distribution pattern, service coverage and redundancy, and centrality correlation. The NNI results are reported in Table 1, Tables 3 and 5, the k-hop coverage and redundancy results in Fig 1, Fig 2, Fig 3, Fig 4, Fig 5, Fig 6, Fig 7, Fig 8, Fig 9, and the Spearman correlation results between functional regions and network centrality in Tables 2, 4 and 6.

**Table 1:** NNI results for save points.

Case	Nearest Neighbour Index	Z score	p-value
**Hollow Knight**	1.302	3.265	p < 0.05
**Blasphemous 2**	1.126	1.487	0.13287
**Prince of Persia: The Lost Crown**	1.095	1.124	0.26973

**Table 2:** Spearman correlation analysis results for Save Point regions and network centrality.

CASE	Spearman’s ρ with Closeness Centrality	p-value	Spearman’s ρ with Betweenness Centrality	p-value
**Hollow Knight**	0.1793	p < 0.05	0.3096	p < 0.05
**Blasphemous 2**	0.8382	p < 0.05	0.4978	p < 0.05
**Prince of Persia: The Lost Crown**	0.2969	p < 0.05	0.3883	p < 0.05

**Table 3:** NNI results for teleport points.

Case	Nearest Neighbour Index	Z score	p-value
**Hollow Knight**	1.372	1.963	p < 0.05
**Blasphemous 2**	1.283	1.860	0.06494
**Prince of Persia: The Lost Crown**	1.149	0.953	0.35664

**Table 4:** Spearman correlation analysis results for Teleporter regions and network centrality.

CASE	Spearman’s ρ with Closeness Centrality	p-value	Spearman’s ρ with Betweenness Centrality	p-value
**Hollow Knight**	0.4714	p < 0.05	0.3917	p < 0.05
**Blasphemous 2**	0.23	p < 0.05	0.1886	p < 0.05
**Prince of Persia: The Lost Crown**	0.7342	p < 0.05	0.4072	p < 0.05

**Table 5:** NNI results for boss points.

Case	Nearest Neighbour Index	Z score	p-value
**Hollow Knight**	1.211	1.489	0.13686
**Blasphemous 2**	1.431	2.390	p < 0.05
**Prince of Persia: The Lost Crown**	1.624	4.615	p < 0.05

**Table 6:** Spearman correlation analysis results for Boss Point regions and network centrality.

CASE	Spearman’s ρ with Closeness Centrality	p-value	Spearman’s ρ with Betweenness Centrality	p-value
**Hollow Knight**	−0.204	p < 0.05	−0.0731	0.1992
**Blasphemous 2**	0.176	p < 0.05	0.0634	0.1306
**Prince of Persia: The Lost Crown**	0.0401	0.5301	0.1079	0.384

### 4.1. Save points

#### 4.1.1. Distribution pattern.

The Nearest Neighbour Index (NNI) values for save points are all greater than 1 across the three games, indicating a dispersed distribution pattern. In Hollow Knight, the save-point NNI is 1.302 (p < 0.05), showing a significant dispersed distribution. In Blasphemous 2, the NNI is 1.126 (p = 0.13287), and in Prince of Persia: The Lost Crown, the NNI is 1.095 (p = 0.26973). Both cases also exhibit a dispersed distribution tendency, although the results are not statistically significant. Overall, the layouts of save points in all three games show a dispersed distribution tendency.

#### 4.1.2. Service coverage and redundancy.

In terms of service coverage, the save-point layouts in all three games show, to varying degrees, stronger coverage tendencies than the random baseline. In other words, within the same hop radius, the probability that a spatial node can reach at least one save point is generally higher than under random placement. This indicates that the save-point configurations in all three cases provide spatial distributions that are more conducive to access to save functionality, although the degree of enhancement differs between cases.

Among the three, Prince of Persia: The Lost Crown shows the clearest coverage advantage. Its coverage curve remains above the random baseline at most scales, especially at small to medium radii. This means that, within the same path range, a larger proportion of spatial nodes can reach a save point earlier, and the overall layout therefore exhibits strong service coverage. Blasphemous 2 also shows a clear tendency toward higher-than-random coverage. Its coverage curve likewise remains above the baseline at most scales, indicating that more spatial nodes can reach save points within a given hop range. Compared with The Lost Crown, however, this enhancement remains slightly weaker in terms of the strength of coverage expansion. In Hollow Knight, the coverage curve generally lies close to, or only slightly above, the random baseline. This suggests that its save-point layout also improves access to save functionality to some extent, but the enhancement is comparatively mild and less pronounced than in the other two cases.

In terms of service redundancy, Blasphemous 2 shows the most evident redundancy tendency. Its redundancy curve lies above the random baseline at most scales, indicating that, within a given path range, spatial nodes are more likely to reach multiple save points at the same time. In other words, more alternative save locations are available within the same range. This suggests a denser spatial relationship among save points, allowing more regions to be covered jointly by multiple save nodes. By contrast, the redundancy level in Prince of Persia: The Lost Crown remains generally close to the random baseline. Its redundancy curve is only slightly above, or close to, the baseline at most scales, indicating that although spatial nodes can usually reach save points within a certain range, the probability of simultaneously reaching multiple save points is not substantially higher than under random placement. Accordingly, the degree of overlap among save points remains limited. The redundancy curve in Hollow Knight is also generally close to the baseline, and at some scales even slightly below it. This indicates that, within the same path range, the probability of simultaneously reaching multiple save points is relatively low, and the service ranges of different save points overlap less strongly.

#### 4.1.3. Centrality correlation.

The coupling between save points and network centrality differs substantially across the three games. In Hollow Knight, the correlations between save points and centrality measures are relatively weak (closeness centrality ρ = 0.1793, p < 0.05; betweenness centrality ρ = 0.3096, p < 0.05). Prince of Persia: The Lost Crown likewise shows relatively weak correlations (closeness centrality ρ = 0.2969; betweenness centrality ρ = 0.3883; both p < 0.05). By contrast, save points in Blasphemous 2 are highly centralized, with a closeness centrality correlation of ρ = 0.8382 (p < 0.05) and a betweenness centrality correlation of ρ = 0.4978 (p < 0.05). This indicates that the save points in Blasphemous 2 are much more strongly coupled with the network core and key paths, and thus more closely resemble a hub-oriented layout. In comparison, the correlations in Hollow Knight and The Lost Crown are clearly weaker. Although save points in these two games still maintain some relationship with the overall structure, they are not as clearly anchored to the network core as in Blasphemous 2.

### 4.2. Teleport points

#### 4.2.1. Distribution pattern.

Teleport points in all three games also show an overall tendency toward dispersed distribution. In Hollow Knight, the teleport-point NNI is 1.372 (p < 0.05), indicating a statistically significant dispersed distribution. In Blasphemous 2, the NNI is 1.283 (p = 0.06494), indicating a near-significant dispersed distribution tendency. In Prince of Persia: The Lost Crown, the NNI is 1.149 (p = 0.35664), showing a non-significant dispersed trend.

#### 4.2.2. Service coverage and redundancy.

In terms of service coverage, the teleport-point layouts show more pronounced differentiation across the three games. This indicates that teleport points form different degrees of accessible spatial range in different cases.

Among them, Prince of Persia: The Lost Crown exhibits the strongest coverage advantage. Its coverage curve remains above the random baseline at most scales, especially at small to medium radii. This indicates that, within the same path range, a larger proportion of spatial nodes can reach a teleport point within a relatively short distance, and that the teleport-point layout therefore forms a wider accessible range overall. By contrast, the coverage performance of Blasphemous 2 shows a gradual increase across scales. At small radii, its coverage remains close to, or below, the random baseline. As the hop range increases, however, the coverage curve rises progressively and exceeds the random baseline at medium scales. This indicates that teleport points in Blasphemous 2 are relatively difficult to access at small scales, but gradually cover more spatial nodes at larger ranges, thereby forming a certain degree of overall coverage capacity. The coverage curve of Hollow Knight remains below, or close to, the random baseline across most scales, especially at small radii where accessibility is relatively low. This suggests that, within the same path range, the proportion of spatial nodes able to reach teleport points is generally low. Its teleport points are therefore relatively sparse in spatial distribution and more difficult to access locally. As the scale expands, coverage gradually approaches the baseline, but the overall configuration still does not show a strongly enhanced coverage structure.

In terms of service redundancy, both Blasphemous 2 and Prince of Persia: The Lost Crown show redundancy curves that remain close to the random baseline at most scales. This indicates that the degree of overlap among teleport points is generally similar to that under random placement, and that their spatial structures exhibit a relatively neutral overlap pattern. By contrast, the redundancy level in Hollow Knight remains below the random baseline across most scales. This indicates that, within the same path range, the probability of spatial nodes simultaneously reaching multiple teleport points is relatively low. Teleport points thus maintain a more dispersed spatial relationship, and the overlap among their service areas remains comparatively limited.

#### 4.2.3. Centrality correlation.

The correlations between teleport-point locations and network centrality are strongest in Prince of Persia: The Lost Crown, intermediate in Hollow Knight, and weakest in Blasphemous 2. In The Lost Crown, teleport points are highly correlated with core positions in the network (closeness centrality ρ = 0.7342; betweenness centrality ρ = 0.4072; both p < 0.05), indicating that they are located in the topological centre of the game space. Hollow Knight shows a moderate degree of coupling (closeness centrality ρ = 0.4714; betweenness centrality ρ = 0.3917; both p < 0.05), whereas Blasphemous 2 shows markedly weaker correlations (closeness centrality ρ = 0.230; betweenness centrality ρ = 0.1886; both p < 0.05). These results indicate that The Lost Crown is more inclined to configure teleport points as central transportation hubs, Hollow Knight places them in relatively central positions but not as the most dominant nodes, and the dedicated teleport points in Blasphemous 2 show the weakest hub-like characteristics within the network.

### 4.3. Boss points

#### 4.3.1. Distribution pattern.

Boss areas in all three games also show a dispersed distribution tendency. Prince of Persia: The Lost Crown has the most dispersed Boss layout (NNI = 1.624, p < 0.05), and Blasphemous 2 likewise shows a statistically significant dispersed distribution (NNI = 1.431, p < 0.05). In Hollow Knight, the dispersed tendency is weaker and not statistically significant (NNI = 1.211, p = 0.13686).

#### 4.3.2. Service coverage and redundancy.

In terms of service coverage, the Boss-point layouts in all three games remain generally close to the random baseline. Across all three cases, the coverage curves rise gradually with increasing scale and approach full coverage at larger ranges, but the differences from the random baseline remain relatively limited at most scales. This indicates that, within a given path range, the probability of spatial nodes reaching Boss points remains broadly comparable to that under random placement.

By contrast, service redundancy shows a highly consistent structural pattern across all three games: redundancy remains below the random baseline at most scales. This means that, within the same hop radius, the probability of simultaneously reaching multiple Boss points is clearly lower than under random placement. The result suggests that Boss points maintain strong spatial separation from one another and that the overlap among their service areas remains low. Such a structure allows different Boss points to form relatively independent accessible regions within the network.

#### 4.3.3. Centrality correlation.

The correlations between Boss areas and network centrality are generally weak. In Hollow Knight, Boss locations are significantly negatively correlated with closeness centrality (ρ = –0.204, p < 0.05), indicating that Boss areas are more likely to occupy relatively peripheral positions in the network. In Prince of Persia: The Lost Crown, Boss areas show no significant correlation with centrality measures. In Blasphemous 2, only closeness centrality shows a weak positive correlation (ρ = 0.176, p < 0.05), whereas betweenness centrality is not significant. Overall, Boss areas do not function as transportation hubs in any of the three games.

## 5. Discussion

Through a comparative analysis of Hollow Knight, Prince of Persia: The Lost Crown, and Blasphemous 2, this study summarizes the layout characteristics of three types of functional nodes. The results show that different functional nodes exhibit distinct structural characteristics in terms of distribution pattern, service coverage and redundancy, and their relationship with network centrality. These differences correspond to different modes of spatial organization and jointly shape the player’s exploratory rhythm and spatial experience.

### 5.1. Save points: Distributed safety net vs. hub-like strongholds

Across the three games, the overall layout of save points exhibits a relatively weak tendency toward dispersed distribution. When considered together with the service coverage results, however, this dispersed tendency still gives rise to a relatively “fine-meshed” safety net at the global level. Compared with the baseline configuration, the actual save-point layouts cover more spatial nodes at small to medium topological scales, making save points easier for players to encounter during movement. Against this shared background, the main difference between cases lies in how this “safety net” is organized across spatial scales.

In Hollow Knight and Prince of Persia: The Lost Crown, the layout of save points takes the form of a spatially distributed safety net. Save functions are relatively evenly spread across different regions, allowing players to encounter save points with a certain degree of regularity during progression and thus to obtain sustained support in the form of healing and checkpoints. At the same time, the number of save points accessible within the same local range is usually limited, service overlap between nodes remains relatively weak, and the relationship with structurally central positions in the network is also comparatively limited. Taken together, these characteristics produce a more typical distributed safety-net structure.

Blasphemous 2, by contrast, presents a different organizational mode on the basis of a similar dispersed tendency. As the largest map among the three cases, it allows players to travel between save points, effectively integrating saving and fast travel into the same system. As a result, save points in Blasphemous 2 are more strongly aligned with topologically central positions and display higher coverage and redundancy at small radii. This gives rise to a “hub-like stronghold” pattern: save points are placed in structurally central positions and exhibit a certain degree of overlap in coverage, forming powerful hub nodes that support repeated return and circulation.

### 5.2. Teleport points: Auxiliary facilities and flow optimization

The teleport-point layouts in all three games exhibit a dispersed distribution tendency, yet their spatial roles differ substantially across cases. The key distinction lies in the extent to which fast movement is allowed to intervene in the player’s exploration process: whether the teleport system functions merely as an auxiliary transport facility, or whether it further develops into a system-level tool for organizing spatial flow and optimizing progression.

Compared with the other cases, teleport points in Hollow Knight function more as exploration-oriented auxiliary transport facilities. Although they are dispersed across different regions, they are not easily accessed at small scales, nor do they form especially strong continuous linkages with one another. Because teleport points in Hollow Knight are deliberately placed in relatively restrained locations, the teleport system is not organized into an easily accessible transport network. Consequently, map progression continues to rely primarily on continuous traversal itself, requiring players to repeatedly pass through routes and re-identify regional relations in order to gradually construct an understanding of the overall space.

Teleport points in Blasphemous 2 are closer to a supplementary auxiliary transport facility. Since part of the fast-movement function is integrated into the save-point system, rapid travel is no longer carried entirely by dedicated teleport points. The structural importance of dedicated teleport points is therefore partially reduced: they still participate in organizing movement, but they are no longer the only transport core, nor the strongest one.

In Prince of Persia: The Lost Crown, teleport points more clearly serve the purpose of flow optimization. As the smallest game world among the three cases, its teleport system as the carrier of fast travel is not fully opened until the later stage of the game. Accordingly, early spatial experience still depends mainly on ordinary traversal. Once the system is activated in the later stage, however, teleport points—being easier to access and located closer to key positions in the game space—can connect multiple regions with relatively high efficiency. This not only substantially reduces the burden of long-distance backtracking and repeated running, but also facilitates the later-stage clearing of previously unresolved exploration content.

### 5.3. Boss points: Isolated challenges and peripheral organization

The spatial layout of Boss points shows a relatively consistent pattern across the three games. In all cases, Boss points are arranged in a dispersed manner, exhibit relatively low overlap in coverage, and are less likely to occupy structurally central positions in the game space. This combination means that Boss encounters are spatially separated from one another and tend to form relatively self-contained challenge units.

Under such a layout, Boss encounters are more easily distinguished from the ordinary rhythm of exploration. Players typically need to go through a relatively explicit process of progression before truly entering the area where a Boss is located. Precisely for this reason, Boss battles are able to retain a strong sense of independence and phase distinction within the broader exploratory process. This kind of layout also helps elevate each Boss encounter into a more autonomous and memorable spatial event—one that often functions as a climax in the player’s overall journey.

## 6. Conclusion

By modeling Metroidvania game spaces as networks, this study provides a preliminary analysis of the layout of key functional nodes in three cases and uses it to compare differences in functional-node layout design across games. At the methodological level, the study attempts to introduce into video game research the analytical logic developed in real-space studies concerning the relationship between spatial structure and functional facilities, while adapting relevant methods to the characteristics of video game space. Specifically, the study replaces Euclidean distance with network shortest-path distance to adapt the Nearest Neighbor Index (NNI), thereby identifying whether functional nodes exhibit clustered, uniform, or dispersed distribution patterns within the overall structure. It also employs graph-based multi-source k-hop coverage and redundancy analysis to examine, against a random-layout baseline, the coverage capacity and degree of service overlap of different types of functional nodes across topological scales. In addition, by testing the relationship between functional regions and centrality, the study examines whether a given type of functional node and its adjacent spaces tend to occupy structurally more central positions in the network. On this basis, the study further interprets the observed data trends and analytical results within the specific design context of game-space organization and functional node layout, in order to discuss the structural implications they may suggest.

At the practical level, this framework can serve as a diagnostic and supportive tool for level design, enabling designers to evaluate and examine the effectiveness of functional node layouts and to determine whether they are consistent with design objectives in terms of coverage, overlap, structural position, and the extent to which functional nodes realize their intended roles. By bringing the analysis back to the level of design intention, it also helps designers assess whether the current layout supports the intended experiential goals and, on this basis, adjust the layout according to specific needs—for example, by reinforcing weak areas, compressing redundant configurations, and re-anchoring key nodes to positions that exert stronger influence on overall movement flow; or, conversely, by deliberately keeping certain functions in relatively restrained positions in order to preserve a sense of exploration, backtracking, and rhythmic tension.

Finally, it should be emphasized that the discussion in this study is based primarily on static topological analysis of space. The current framework does not model mechanisms that are dynamically unlocked over the course of play, nor does it incorporate key spatial-semantic factors such as resource nodes or narrative elements. Because such factors are closely tied to player motivation for exploration and real-time path selection, the present results are better suited to explaining the relationship between functional layout and spatial structure than to fully capturing the player’s complete gameplay experience. At the same time, the number of cases examined remains limited. The present discussion should therefore be regarded primarily as an interpretive framework and a methodological demonstration.

Future research may proceed in three directions. First, it may move from static analysis to dynamic analysis by dividing the gameplay process into different stages and examining how the spatial role of functional nodes changes under different progression states. Second, it may introduce the analysis of spatial semantics in order to more accurately capture the logic underlying functional-node placement. Third, it may integrate player-behavior data—such as movement trajectories and death locations—and relate these data to spatial models, thereby constructing a more comprehensive analytical framework. As this framework is further developed, it is hoped that network analysis will gradually develop into a more comprehensive research approach for linking spatial structure, design decisions, and player experience.

## Supporting information

S1 Table Full k-hop coverage and redundancy results for Hollow Knight. (XLSX)

S2 Table Full k-hop coverage and redundancy results for Blasphemous 2. (XLSX)

S3 Table Full k-hop coverage and redundancy results for Prince of Persia: The Lost Crown. (XLSX)
